# Persistently short or long sleep duration increases the risk of sensory impairment in Chinese older adults

**DOI:** 10.3389/fpubh.2024.1329134

**Published:** 2024-02-29

**Authors:** Ling Yang, Jing Du, Ying Duan, Yan Cui, Qi Qi, Zihao Liu, Huaqing Liu

**Affiliations:** School of Public Health, Bengbu Medical University, Bengbu, Anhui, China

**Keywords:** sleep duration, altered sleep duration, sensory impairment, hearing impairment, visual impairment, CLHLS, older adults

## Abstract

**Background:**

Cross-sectional evidence suggests that persistently short or long sleep duration is associated with sensory impairment. Thus, this study was conducted to investigate the associations between sleep duration and altered sleep duration with sensory impairment in Chinese older adults.

**Methods:**

Longitudinal data (2008–2014) obtained through the Chinese Longitudinal Healthy Longevity Survey (CLHLS) were analyzed. Sleep duration was classified as normal (7–8 h), short (<7 h), or long (≥9 h). Sensory impairment was assessed using individuals’ self-reported data on visual and hearing functions. Cox regression was performed to evaluate the effects of sleep duration and altered sleep duration on sensory impairment, including visual impairment (VI) and hearing impairment (HI).

**Results:**

This study included 3,578 older adults (mean age: 78.12 ± 9.59 years). Among them, 2,690 (75.2%) were aged 65–84 years and 1798 (50.3%) were women. The risks of VI (hazard ratio [HR]: 1.14; 95% confidence interval [CI]: 1.02–1.29), HI (HR: 1.14; 95% CI: 1.00–1.30), and dual sensory impairment (both VI and HI; HR: 1.26; 95% CI: 1.03–1.55) were high in older adults with long sleep duration. In addition, the risks of VI, HI, and dual sensory impairment were high in individuals whose sleep duration changed from normal to short or long (HR: 1.20 [95% CI: 1.02–1.42], 1.26 [95% CI: 1.03–1.53], and 1.54 [95% CI: 1.11–2.12], respectively) and those with persistently short or long sleep duration (HR: 1.25 [95% CI: 1.07–1.46], 1.34 [95% CI: 1.11–1.61], and 1.67 [95% CI: 1.22–2.27], respectively).

**Conclusion:**

A prospective association was identified between altered sleep duration and sensory impairment in Chinese older adults. Our findings highlight the importance of optimal sleep duration and healthy sleep habits in preventing sensory impairment in older adults.

## 1 Introduction

Aging is associated with several disorders that compromise individuals’ quality of life. Among these disorders, sensory impairment—visual impairment (VI) and hearing impairment (HI)—are common in older adults. The severity of these disorders increase with age (1). The concurrent occurrence of VI and HI is termed as dual sensory impairment (DSI). DSI prevents individuals from compensating for one lost sense with another, which makes communication difficult for these individuals (2). DSI affects >10% of all older adults worldwide (3). Sensory impairment is associated with elevated risks of mortality (3), depression (4), dementia (5), anxiety, and cognitive impairment (1); increased use of health-care resources; and increased health expenditure (6).

Sleep duration is a key factor that influences normal physiological functions. The amount of sleep required for optimal functioning varies across individuals and life stages. A daily sleep duration of 7–8 h is recommended for older individuals (7). Sleep duration influences the risks of cognitive dysfunction (8), depression (9), and reduced muscle strength (10).

Suboptimal sleep duration may lead to sensory impairment in older adults. A U-shaped association has been observed between sleep duration and VI (11). Both sleep deprivation and excessive sleep are associated with VI (12). A study conducted among Chinese individuals indicated that a daily sleep duration ≥8 h is significantly associated with HI (13). Similarly, a study involving 632 individuals aged ≥70 years reported that a sleep duration of >8 h is associated with increased hearing thresholds (14). Another cross-sectional survey conducted among Chinese older adults revealed that a short sleep duration is associated with increased risks of VI, HI, and DSI (15).

Most studies investigating the association of short or long sleep duration with sensory impairment have had a cross-sectional design; few studies have demonstrated a longitudinal association between altered sleep duration and sensory impairment. Thus, we conducted this study to investigate the associations of sleep duration and altered sleep duration with sensory impairment in Chinese older adults. The analysis was performed using longitudinal data (2008–2014) collected through the Chinese Longitudinal Healthy Longevity Survey (CLHLS).

## 2 Methods

### 2.1 Study cohort

CLHLS is an ongoing prospective follow-up survey of longevity and health among Chinese older adults. This survey was conducted in 23 randomly selected cities and counties across 31 Chinese provinces including approximately 85% of the total population of China. Approval for the use of CLHLS data was obtained from the Ethics Committee of Peking University (IRB00001052-13074). Written informed consent was obtained from all participants or their representatives. Further details of the CLHLS data set can be found elsewhere (16–18).

**Figure 1:**
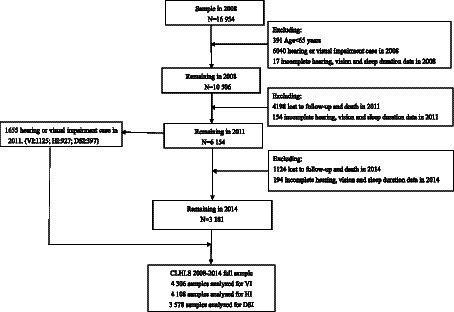
Flowchart depicting participant selection.

We analyzed longitudinal survey data that were conducted in 2008 to 2014, including three surveys in 2008, 2011, and 2014. Figure 1 depicts the participant selection process. In the 2008 survey, 16,954 individuals participated in baseline interviews. We excluded individuals aged <65 years (*n* = 391); those with sensory impairment (including HI or VI) (*n* = 6,040); those with incomplete hearing, vision, or sleep duration data in the 2008 survey (*n* = 17); and those who were lost to follow-up, died, or had incomplete hearing, vision, or sleep duration data in the 2011 survey (*n* = 4,352). By the 2011 survey, 6,154 participants—1,125 with VI, 927 with HI, and 397 with DSI—remained eligible for inclusion in this study. We identified 4,499 participants who did not have sensory impairment in 2011. We further excluded participants who were lost to follow-up or died by the 2014 survey and those with incomplete hearing, vision, or sleep duration data in the 2014 survey (*n* = 1,318). Thus, 3,181 participants remained eligible for inclusion in this study. Finally, 4,306, 4,138, and 3,578 participants were included in the analyses of VI, HI, and DSI, respectively.

### 2.2 Assessment of sleep duration

Sleep duration was evaluated on the basis of the participants’ responses to the following question (19): “For how long did you sleep per day over the previous week (excluding daytime sleep and naps)?” On the basis of the National Sleep Foundation–recommended sleep duration for older adults (7), sleep duration was classified as normal (7–8 h), short (<7 h), or long (≥9 h). Furthermore, on the basis of altered sleep duration from 2008 to 2011 or 2014, we classified sleep duration as persistently normal, normal to short or long, short or long to normal, or persistently short or long (4).

### 2.3 Assessment of sensory impairment

Regarding sensory impairment, we focused mainly on HI and VI. On the basis of how well they could hear the questions asked by the CLHLS interviewer, participants were divided into the following four categories: can hear but require a hearing aid; can hear and do not require a hearing aid; can partially hear and require a hearing aid; and cannot hear, even with a hearing aid. Participants belonging to the latter two categories were considered to have HI. Furthermore, on the basis of how well they could see a circle on a card placed 1 m away and discern the direction of a break in the circle after their vision aids (e.g., glasses) were removed, participants were divided into the following four categories: can see the circle and discern the direction of the break; can see the circle but cannot discern the direction of the break; cannot see the circle; and blind. Participants belonging to the latter two categories were considered to have VI (18). Thus, participants were stratified by sensory impairment (20) into the following four groups: no sensory impairment, HI, VI, or DSI.

### 2.4 Covariates

Sensory impairment in older adults can be influenced by individual heterogeneity, socioeconomic level, and health (21). To mitigate the effects of confounders, we adjusted our statistical models for the following covariates: sex (male or female), age (65–84 or ≥ 85 years), ethnicity (Han Chinese or others), marital status (married or unmarried), residential area (urban, township, or rural), cohabitation status (living with family, alone, or in a nursing home), years of education (<1 or ≥ 1 year), alcohol consumption (yes or no), exercise habit (yes or no), smoking status (yes or no), body mass index (BMI) rounded to the nearest 0.1 kg/m^2^ (underweight with a BMI of <18.5 kg/m^2^; normal with a BMI of 18.5–23.9 kg/m^2^; overweight with a BMI of 24–27.9 kg/m^2^; and obese with a BMI of ≥28 kg/m^2^), chronic disease status (yes or no), and sleep quality (good, neutral, or poor). The chronic diseases mainly included hypertension, heart disease, diabetes, arthritis, pneumonia, bronchitis, asthma, emphysema, stroke or cardiovascular disease, gastric or duodenal ulcer, dementia, and cancer.

### 2.5 Statistical analysis

All individuals were stratified by sleep duration. The chi-square test was performed for the between-group comparison of baseline characteristics. Hazard ratios (HRs) with 95% confidence intervals (CIs) for the risks of HI, VI, and DSI were calculated using Cox proportional hazards models. The Schoenfeld residual test was performed to validate the proportional hazard assumption. The final models were adjusted for all of the aforementioned covariates. For individuals who did not have sensory impairment at baseline (in 2008) but had it in 2011, a 3-year interval was considered in the corresponding model. For individuals who did not have sensory impairment in 2008 and 2011 but had it in 2014, a 6-year interval was considered in the corresponding model.

To enhance the robustness of our findings, we conducted several sensitivity analyses. First, we performed multiple imputations on the basis of five replications and Monte Carlo simulations to account for missing data and re-evaluated the association of altered sleep duration with sensory impairment. Second, to adjust for the confounding effects of health-related factors, we restricted the analysis to only individuals with no chronic disease cataract and re-evaluated the aforementioned association. Finally, we analyzed the results after excluding participants who reported extreme sleep duration (<3 or > 16 h) at least once in all three survey waves.

All statistical analyses were performed using SPSS (version 26.0) or R (version 4.2.2). A two-sided *p*-value of <0.05 indicated significance.

## 3 Results

### 3.1 Cohort characteristics

Table 1 presents the baseline characteristics of the study cohort. This study included 3,578 older adults (mean age: 78.12 ± 9.59 years). Among participants, 1780 (49.7%) were men, 2,690 (75.2%) were aged 65–84 years, 1,312 (36.6%) lived in urban areas or townships, 1989 (55.6%) were married, 2,999 (83.8%) lived with family members, 1741 (48.8%) had received <1 year of education, and 1719 (48.0%) had no chronic disease.

**Table 1:** Baseline characteristics of older adults stratified by sleep duration.

Baseline characteristic	N (%)	Sleep duration			χ^2^	Change in sleep duration from 2008 to 2014				χ^2^
		7-8 h N (%)	<7 h N (%)	≥9 h N (%)		Persistent normal	Change from normal into short or long N (%)	Change from short or long into normal N (%)	Persistent short or long N (%)	
Total	3,578	1,598 (44.7)	950 (26.6)	1,030 (28.8)		664 (18.6)	934 (26.1)	660 (18.4)	1,320 (36.9)	
Age					59.54***					56.57***
65–84	2,690 (75.2)	1,246 (46.3)	759 (28.2)	685 (25.5)		557 (20.7)	689 (25.6)	525 (19.5)	919 (34.2)	
≥85	888 (24.8)	352 (439.6)	191 (21.5)	345 (38.9)		107 (12.0)	245 (27.6)	135 (15.2)	401 (45.2)	
Gender					11.74**					2.70
Male	1780 (49.7)	794 (44.6)	435 (24.4)	551 (31.0)		338 (19.0)	456 (25.6)	338 (19.0)	648 (36.4)	
Female	1798 (50.3)	907 (45.0)	590 (29.3)	520 (25.8)		326 (18.1)	478 (26.6)	322 (17.9)	672 (37.4)	
Ethnicity					0.36					2.32
Han	3,345 (93.5)	1,486 (44.4)	897 (26.8)	962 (28.8)		616 (18.4)	870 (26.0)	625 (18.7)	1,234 (36.9)	
Others	223 (6.5)	1,112 (48.1)	53 (22.7)	68 (29.2)		48 (20.6)	64 (27.5)	35 (15.0)	86 (36.9)	
Residence					29.60***					4.23
Urban	545 (15.2)	256 (47.0)	175 (32.1)	114 (20.9)		113 (20.7)	143 (26.2)	99 (18.2)	190 (34.92)	
Town	767 (21.4)	343 (44.7)	170 (22.2)	254 (33.1)		131 (17.1)	212 (27.6)	137 (17.9)	287 (37.4)	
Rural	2,266 (63.3)	999 (41.4)	605 (26.7)	662 (29.2)		420 (18.5)	579 (25.6)	424 (18.7)	843 (37.2)	
Marital status					11.38**					23.50***
Married	1989 (55.6)	924 (46.5)	537 (27.0)	528 (26.5)		411 (20.7)	513 (25.8)	388 (19.5)	677 (34.0)	
Others	1,589 (44.4)	674 (42.4)	413 (26.0)	502 (31.6)		253 (15.9)	421 (26.5)	272 (17.1)	643 (40.5)	
Co-residence					1.45					26.20***
With family	2,999 (83.8)	1,345 (44.8)	793 (26.4)	861 (28.7)		586 (19.5)	759 (25.3)	575 (19.2)	1,079 (36.0)	
Alone	543 (15.2)	236 (43.5)	150 (27.6)	157 (28.9)		75 (13.8)	161 (29.7)	82 (15.1)	225 (41.4)	
In institution	36 (1.0)	17 (47.2)	7 (19.4)	12 (33.3)		3 (8.3)	14 (38.9)	3 (8.3)	16 (44.4)	
The years of education					12.24**					24.89***
<1 year	1741 (48.8)	749 (43.0)	443 (25.4)	549 (31.5)		276 (15.9)	473 (27.2)	300 (17.2)	692 (39.7)	
≥1 year	1830 (51.2)	749 (43.0)	443 (25.4)	549 (31.5)		385 (21.0)	461 (25.2)	360 (19.7)	624 (34.1)	
Smoking at present					1.18					7.48
Yes	842 (23.5)	363 (43.1)	233 (27.7)	246 (29.2)		148 (17.6)	215 (25.5)	182 (21.6)	297 (35.3)	
No	2,736 (76.5)	1,235 (45.1)	717 (26.2)	784 (28.7)		516 (18.9)	719 (26.3)	478 (17.5)	1,023 (37.4)	
Drinking at present					0.83					1.33
Yes	792 (22.1)	362 (45.7)	212 (26.8)	218 (27.5)		158 (19.9)	204 (25.8)	142 (17.9)	288 (36.4)	
No	2,786 (77.9)	1,236 (44.4)	738 (26.5)	812 (29.1)		506 (18.2)	730 (26.2)	518 (18.6)	1,032 (37.0)	
Exercise at present					12.72^***^					11.62^***^
Yes	1,330 (37.2)	615 (46.2)	378 (28.4)	337 (25.3)		261 (19.6)	354 (26.6)	269 (20.2)	446 (33.5)	
No	2,248 (62.8)	983 (43.7)	572 (25.4)	693 (30.8)		403 (17.9)	580 (25.8)	391 (17.4)	874 (38.9)	
BMI (kg/m^2^)					7.14					10.13
<18.5	753 (21.1)	340 (45.2)	215 (28.6)	198 (26.3)		129 (17.1)	211 (28.0)	128 (17.0)	285 (37.8)	
18.5–23.9	2090 (58.6)	944 (45.2)	534 (25.6)	612 (29.3)		395 (18.9)	549 (26.3)	396 (18.9)	750 (35.9)	
24–27.9	569 (16.0)	244 (42.9)	163 (28.6)	162 (28.5)		104 (18.3)	140 (24.6)	109 (19.2)	216 (38.0)	
≥28	154 (4.3)	65 (42.2)	37 (24.0)	52 (33.8)		35 (22.7)	30 (19.5)	25 (16.2)	64 (41.6)	
Number of chronic diseases					61.31^***^					8.57^*^
0	1719 (48.0)	792 (46.1)	359 (20.9)	568 (33.0)		325 (18.9)	467 (27.2)	334 (19.4)	593 (34.5)	
≥1	1809 (50.6)	789 (43.6)	573 (31.7)	447 (24.7)		332 (18.4)	457 (25.3)	313 (17.3)	707 (39.1)	
Sleep quality					1076.58^***^					200.64^***^
Good	2,454 (68.6)	1,233 (50.2)	291 (11.9)	930 (37.9)		518 (21.1)	715 (29.1)	415 (16.9)	806 (32.8)	
Neutral	783 (21.9)	325 (41.5)	376 (48.0)	82 (10.5)		138 (17.6)	187 (23.9)	166 (21.2)	292 (37.3)	
Poor	340 (9.5)	39 (11.5)	283 (83.2)	18 (5.3)		8 (2.4)	31 (9.1)	79 (23.2)	222 (65.3)	

*p < 0.05, **p < 0.01, and ***p < 0.001.

Persistently normal sleep duration was noted in individuals who were aged 65–84 years, were married, lived with family members, received formal education, exercised (during the period around the CLHLS), or had no chronic disease.

### 3.2 Association between sleep duration and sensory impairment

This study included 1,606 (37.3%) individuals with VI, 1239 (30.2%) individuals with HI, and 522 (14.6%) individuals with DSI. Table 2 presents the incidence rates of different forms of sensory impairment due to altered sleep duration. The incidence rates of VI, HI, and DSI were the lowest for individuals with persistently normal sleep duration (28.8, 20, and 7.8%, respectively) but the highest for those with persistently short or long sleep duration (42.0, 36.4, and 18.8%, respectively).

**Table 2:** Incidence of sensory impairment due to altered sleep duration.

Characteristics	Visual functions			Hearing functions			Visual and hearing functions		
	*N* (%)	VI *n* (%)	χ^2^	*N* (%)	HI *n* (%)	χ^2^	*N* (%)	DSI *n* (%)	χ^2^
Total	4,306 (100)	1,606 (37.3)		4,108	1,239 (30.2)		3,578	552 (14.6)	
Sleep duration change category			44.01^***^			76.88***			48.19***
Persistent normal	774 (18.0)	223 (28.8)		726 (17.7)	145 (20.0)		664 (18.6)	52 (7.8)	
Change from normal into short or long	1,123 (26.1)	434 (38.6)		1,089 (26.5)	347 (31.9)		934 (26.1)	145 (15.5)	
Change from short or long into normal	789 (18.3)	268 (34.0)		734 (17.9)	180 (24.5)		660 (18.4)	77 (11.7)	
Persistent short or long	1,620 (37.6)	681 (42.0)		1,559 (38.0)	567 (36.4)		1,320 (36.9)	248 (18.8)	
Sleep duration at baseline			20.48***			57.7***			47.62***
7-8 h	1897 (44.1)	657 (34.6)		1815 (44.2)	492 (27.1)		1,598 (44.7)	197 (12.3)	
<7 h	1,147 (26.6)	414 (36.1)		1,075 (26.2)	278 (25.9)		950 (26.6)	109 (11.5)	
≥9 h	1,262 (29.3)	535 (42.4)		1,218 (29.6)	469 (38.5)		1,030 (28.8)	216 (21.0)	

*p < 0.05, **p < 0.01, and ***p < 0.001.

The risks of VI (HR: 1.14; 95% CI: 1.02–1.29), HI (HR: 1.14; 95% CI: 1.00–1.30), and DSI (HR: 1.26; 95% CI: 1.03–1.55) were significantly higher in individuals with long sleep durations than in those with normal sleep durations (Table 3). In addition, the risks of VI, HI, and DSI were significantly higher in individuals whose sleep durations changed from normal to short or long (HR: 1.20 [95% CI: 1.02–1.42], 1.26 [95% CI: 1.03–1.53], and 1.54 [95% CI: 1.11–2.12], respectively) and those with persistently short or long sleep duration (HR: 1.25 [95% CI: 1.07–1.46], 1.34 [95% CI: 1.11–1.61], and 1.67 [95% CI: 1.22–2.27], respectively) than in those with persistently normal sleep duration.

**Table 3:** Multivariate cox regression models for the association between baseline sleep duration and sensory impairment.

Characteristics	Crude model HR (95% CI)			Final model HR (95% CI)		
	VI	HI	DSI	VI	HI	DSI
Sleep duration change category (ref. = Persistent normal)	1.00	1.00	1.00	1.00	1.00	1.00
Change from normal into short or long	1.37 (1.17–1.61) ^***^	1.65 (1.36–2.00) ^***^	2.01 (1.46–2.76) ^***^	1.20 (1.02–1.42) ^*^	1.26 (1.03–1.53) ^*^	1.54 (1.11–2.12) ^*^
Change from short or long into normal	1.20 (1.00–1.43) ^*^	1.24 (1.00–1.55)	1.50 (1.06–2.14) ^*^	1.15 (0.96–1.38)	1.12 (0.90–1.40)	1.41 (0.99–2.02)
Persistent short or long	1.52 (1.31–1.77) ^***^	1.90 (1.59–2.28) ^***^	2.45 (1.82–3.31) ^***^	1.25 (1.07–1.46) ^**^	1.34 (1.11–1.61) ^**^	1.67 (1.22–2.27) ^**^
Sleep duration at baseline (ref. = 7–8)	1.00	1.00	1.00	1.00	1.00	1.00
<7	1.05 (0.92–1.18)	0.95 (0.82–1.10)	0.93 (0.74–1.17)	0.99 (0.86–1.14)	1.00 (0.84–1.18)	1.02 (0.78–1.34)
≥9	1.27 (1.13–1.42) ^***^	1.47 (1.29–1.66) ^***^	1.74 (1.43–2.11) ^***^	1.14 (1.02–1.29) ^*^	1.14 (1.00–1.30)	1.26 (1.03–1.55) ^*^

*p < 0.05, **p < 0.01, and ***p < 0.001.

VI, visual impairment; HI, hearing impairment; DSI, dual sensory impairment; and ref., reference.

The final model was adjusted for age, sex, ethnicity, residential area, marital status, cohabitation status, years of education, smoking habit, alcohol consumption, exercise habit, body mass index, chronic disease status, and sleep quality.

### 3.3 Results of subgroup analysis

Our stratified analysis (Table 4) revealed the associations of long sleep duration with elevated risks of VI (HR: 1.24; 95% CI: 1.02–1.50), HI (HR: 1.30; 95% CI: 1.03–1.64), and DSI (HR: 1.49; 95% CI: 1.01–2.19) in individuals with ≥1 year of education. Furthermore, strong associations were noted between persistently short or long sleep duration and elevated risks of VI, HI, and DSI in younger older adults (HR: 1.32 [95% CI: 1.07–1.62], 1.51 [95% CI: 1.11–2.06], and 2.07 [95% CI: 1.18–3.66], respectively), individuals with ≥1 year of education (HR: 1.33 [95% CI: 1.05–1.70], 1.65 [95% CI: 1.19–2.29], and 2.38 [95% CI: 1.30–4.38], respectively), and unmarried individuals (HR: 1.26 [95% CI: 1.02–1.55], 1.31 [95% CI: 1.04–1.66], and 1.49 [95% CI: 1.04–2.14], respectively).

**Table 4:** Results of a subgroup analysis for the association between altered sleep duration and sensory impairment.

Subgroup analysis	Characteristics	VI	HI	DSI
		HR (95% CI)	HR (95% CI)	HR (95% CI)
Age	Sleep duration change category (ref. = Persistent normal)	1.00	1.00	1.00
65–84	Change from normal into short or long	1.24 (1.00–1.54)	1.42 (1.03–1.96) ^*^	1.95 (1.09–3.49) ^*^
	Change from short or long into normal	1.08 (0.85–1.37)	1.14 (0.79–1.63)	1.28 (0.66–2.51)
	Persistent short or long	1.32 (1.07–1.62) ^*^	1.51 (1.11–2.06) ^*^	2.07 (1.18–3.66) ^*^
	Sleep duration at baseline (ref. = 7–8)	1.00	1.00	1.00
	<7	0.99 (0.82–1.97)	0.92 (0.69–1.22)	1.04 (0.62–1.66)
	≥9	1.16 (0.98–1.38)	1.25 (0.98–1.58)	1.25 (0.84–1.88)
≥85	Sleep duration change category (ref. = Persistent normal)	1.00	1.00	1.00
	Change from normal into short or long	1.15 (0.89–1.49)	1.13 (0.88–1.46)	1.36 (0.92–2.01)
	Change from short or long into normal	1.22 (0.92–1.62)	1.08 (0.81–1.43)	1.44 (0.94–2.20)
	Persistent short or long	1.17 (0.91–1.49)	1.20 (0.95–1.53)	1.51 (1.04–2.19) ^*^
	Sleep duration at baseline (ref. = 7–8)	1.00	1.00	1.00
	<7	0.97 (0.77–1.21)	1.03 (0.83–1.26)	1.03 (0.75–1.43)
	≥9	1.12 (0.95–1.32)	1.09 (0.93–1.28)	1.27 (1.00–1.62) ^*^
The years of education	Sleep duration change category (ref. = Persistent normal)	1.00	1.00	1.00
<1 year	Change from normal into short or long	1.20 (0.97–1.49)	1.19 (0.94–1.52)	1.42 (0.97–2.07)
	Change from short or long into normal	1.25 (0.98–1.59)	1.06 (0.80–1.40)	1.42 (0.94–2.16)
	Persistent short or long	1.21 (0.98–1.50)	1.20 (0.94–1.50)	1.45 (1.01–2.09) ^*^
	Sleep duration at baseline (ref. = 7–8)	1.00	1.00	1.00
	<7	1.05 (0.88–1.26)	0.95 (0.77–1.17)	1.03 (0.75–1.42)
	≥9	1.10 (0.94–1.28)	1.06 (0.91–1.25)	1.18 (0.93–1.50)
≥1 year	Sleep duration change category (ref. = Persistent normal)	1.00	1.00	1.00
	Change from normal into short or long	1.20 (0.92–1.53)	1.40 (0.99–1.97)	1.97 (1.05–3.70) ^*^
	Change from short or long into normal	1.01 (0.77–1.34)	1.25 (0.86–1.83)	1.42 (0.70–2.89)
	Persistent short or long	1.33 (1.05–1.70) ^*^	1.65 (1.19–2.29) ^**^	2.38 (1.30–4.38) ^**^
	Sleep duration at baseline (ref. = 7–8)	1.00	1.00	1.00
	<7	0.90 (0.72–1.14)	1.08 (0.81–1.44)	0.97 (0.58–1.62)
	≥9	1.24 (1.02–1.50) ^*^	1.30 (1.03–1.64) ^*^	1.49 (1.01–2.19) ^*^
Marital status	Sleep duration change category (ref. = Persistent normal)	1.00	1.00	1.00
Married	Change from normal into short or long	1.22 (0.95–1.57)	1.28 (0.92–1.79)	1.77 (0.94–3.315)
	Change from short or long into normal	1.11 (0.84–1.46)	0.99 (0.68–1.47)	1.189 (0.57–2.49)
	Persistent short or long	1.26 (0.99–1.61)	1.38 (1.00–1.88)	2.22 (1.20–4.08) ^*^
	Sleep duration at baseline (ref. = 7–8)	1.00	1.00	1.00
	<7	1.00 (0.80–1.25)	0.88 (0.65–1.20)	0.90 (0.52–1.54)
	≥9	1.13 (0.93–1.37)	1.18 (0.93–1.51)	1.50 (1.00–2.24) ^*^
others	Sleep duration change category (ref. = Persistent normal)	1.00	1.00	1.00
	Change from normal into short or long	1.20 (0.96–1.49)	1.23 (0.97–1.58)	1.45 (1.00–2.12) ^*^
	Change from short or long into normal	1.18 (0.93–1.51)	1.17 (0.89–1.54)	1.46 (0.97–2.21)
	Persistent short or long	1.26 (1.02–1.55) ^*^	1.31 (1.04–1.66) ^*^	1.49 (1.04–2.14) ^*^
	Sleep duration at baseline (ref. = 7–8)	1.00	1.00	1.00
	<7	0.99 (0.83–1.19)	1.04 (0.86–1.28)	1.07 (0.78–1.46)
	≥9	1.16 (0.99–1.34)	1.12 (0.95–1.31)	1.18 (0.93–1.49)

*p < 0.05, **p < 0.01, and ***p < 0.001.

VI, visual impairment; HI, hearing impairment; DSI, dual sensory impairment; and ref., reference.

The final model was adjusted for age, sex, ethnicity, residential area, marital status, cohabitation status, years of education, smoking habit, alcohol consumption, exercise habit, body mass index, chronic disease status, and sleep quality.

### 3.4 Results of sensitivity analyses

Sensitivity analyses (Table 5) were performed for different situations, such as accounting for missing data with multiple interpolation, assessing older adults without any major chronic diseases or cataract, and excluding individuals with extreme sleep durations. The associations of sleep duration and altered sleep duration with sensory impairment remained consistent across the analyses.

**Table 5:** Results of a sensitivity analysis for the association between altered sleep duration and sensory impairment.

Outcomes	VI, HR (95% CI)			HI, HR (95% CI)			DSI, HR (95% CI)		
	Change from normal into short or long	Change from short or long into normal	Persistent short or long	Change from normal into short or long	Change from short or long into normal	Persistent short or long	Change from normal into short or long	Change from short or long into normal	Persistent short or long
1.Dealing with the missing data by multiple interpolation	1.21 (1.03–1.43) ^*^	1.16 (0.97–1.38)	1.26 (1.07–1.47) ^**^	1.26 (1.04–1.53) ^*^	1.14 (0.91–1.41)	1.33 (1.11–1.61) ^**^	1.55 (1.12–2.14) ^**^	1.41 (0.98–2.03)	1.66 (1.22–2.26) ^**^
2.Individuals without chronic diseases at baseline	1.23 (0.97–1.57)	1.18 (0.91–1.53)	1.36 (1.08–1.71) ^**^	1.22 (0.93–1.61)	1.09 (0.81–1.49)	1.38 (1.06–1.78) ^*^	0.60 (0.40–0.90) ^*^	0.88 (0.67–1.16)	0.76 (0.54–1.07)
3.Individuals without cataracts at baseline	1.25 (1.05–1.48) ^*^	1.14 (0.94–1.38)	1.30 (1.10–1.54) ^**^	1.32 (1.08–1.62) ^**^	1.14 (0.91–1.45)	1.39 (1.14–1.69) ^**^	1.66 (1.17–2.33) ^*^	1.45 (0.99–2.13)	1.82 (1.31–2.54) ^***^
4.Excluding individuals who reported extreme sleep duration (< 3 or > 16 h)	1.20 (1.01–1.41) ^*^	1.14 (0.95–1.37)	1.24 (1.06–1.45) ^**^	1.25 (1.03–1.53) ^*^	1.13 (0.91–1.42)	1.34 (1.11–1.62) ^**^	1.53 (1.10–2.12) ^*^	1.44 (1.01–2.07)	1.67 (1.22–2.29) ^**^

*p < 0.05, **p < 0.01, and ***p < 0.001.

VI, visual impairment; HI, hearing impairment; DSI, dual sensory impairment. Reference group: individuals with persistently normal sleep duration.

The final model was adjusted for age, sex, ethnicity, residential area, marital status, cohabitation status, years of education, smoking habit, alcohol consumption, exercise habit, body mass index, chronic disease status, and sleep quality.

## 4 Discussion

In this large longitudinal study, we investigated the prospective effects of altered sleep duration on sensory impairment in individuals aged ≥65 years. Our findings revealed a high incidence of sensory impairment in these individuals. Furthermore, sensory impairment was associated with sleep duration and altered sleep duration. The incidence of sensory impairment was the lowest for persistently normal sleep duration. Compared with normal sleep duration, a sleep duration of ≥9 h was associated with an elevated risk of sensory impairment. During the 6-year follow-up period, the risk of sensory impairment was higher in individuals with persistently short or long sleep duration and those with changes from normal to short or long sleep duration than in individuals with persistently normal sleep duration. We further analyzed the effects of altered sleep duration on sensory impairment and obtained new evidence for preventing or delaying the development of sensory impairment in older adults.

A survey of Chinese individuals aged >60 years revealed that the prevalence of VI, HI, and DSI was 80.2, 64.9, and 57.2%, respectively (22). The discrepancy between these values and those estimated in our study may be attributable to the following facts: we analyzed only new cases of sensory impairment recorded between 2008 and 2014; moreover, we reported the incidence of sensory impairment, whereas the aforementioned survey reported its prevalence.

In general, individuals who sleep for 7 to 8 h have better sensory function, lower morbidity risks, and better quality of life than do those who do not (15, 23, 24). A systematic review indicated that long sleep duration is associated with an elevated risk of neurodegenerative disease (25). Both short and long sleep durations can lead to cognitive impairment (8, 26), which is strongly correlated with sensory impairment (27). Therefore, sleep duration may influence the risk of sensory impairment. Several studies have demonstrated that short and long sleep durations contribute to the development of sensory impairment (11, 12, 14, 15). We found that a sleep duration of ≥9 h is associated with an elevated risk of sensory impairment.

Most studies on sleep and sensory impairment have adopted a cross-sectional approach; few longitudinal studies have investigated the effects of sleep duration and altered sleep duration on sensory impairment in older adults. These individuals may experience changes in their sleep patterns, circadian rhythms, sleep self-balancing mechanisms, and levels and patterns of sleep-related hormone secretion (28). Thus, sleep duration changes with age. A cohort study revealed an association between altered sleep duration and depression (4). Depression is associated also with sensory impairment (29, 30). In this study, we hypothesized that altered sleep duration would influence the risk of sensory impairment. To confirm the hypothesis, we longitudinally investigated the effects of sleep duration and altered sleep duration on sensory impairment. Our findings indicate that, compared with persistently normal sleep duration, persistently short or long sleep duration and changes from normal to short or long sleep duration are associated with elevated risks of sensory impairment.

Although the precise mechanisms underlying the effects of sleep duration on sensory impairment remain unclear, their association may be explained as follows. First, the association may be attributable to age-related changes in sleep patterns, such as reduced nighttime sleep duration, frequent daytime napping, and frequent and prolonged nocturnal awakenings (28). Variations in sleep patterns may result in a substantial disparity between objectively measured and self-reported sleep durations among older adults, leading to the overestimation of self-reported sleep duration (28). Variations in sleep patterns are associated with sleep-related hormone secretion, substantial changes in the brain, sensory processing, and poor quality of life (28). Altered sleep patterns can disrupt energy metabolism (31), leading to lipid deposition in the cochlear basement membrane, thereby causing HI (14, 32, 33). Second, homeostatic regulation of sleep duration influences circadian rhythm (34). Therefore, altered sleep duration may disrupt circadian rhythm, accelerating sensory impairment. Third, altered sleep duration may be indirectly associated with sensory impairment. Excessive sleep contributes to diabetes and cardiovascular disease, which are risk factors for VI and HI (35–38). Older adults with sensory impairment have limited activity and natural light exposure, which can reduce melatonin secretion from the pineal gland (39). Low melatonin levels increase the risks of depressive symptoms and depression, as these conditions are associated with insomnia and daytime sleepiness (40).

Our subgroup analysis indicated that among older adults with a sleep duration of ≥9 h and those with persistently short or long sleep duration, the risk of sensory impairment was higher in individuals with ≥1 year of education than in those with <1 year of education. This finding may be explained by the fact that educated older people are more likely to have stable employment before retirement than are uneducated older people. However, when educated older adults retire, their social status decreases and their social sphere shrinks (17). Older adults with weaker social networks are less active socially, which increases the likelihood of excessive sleep duration (41). Having a weak social network serves as a predictor of excessive sleep (42). If postretirement psychological changes are not addressed in a timely manner, this may lead to depression, which increases the risk of sensory impairment (29).

A longitudinal study on aging in the United Kingdom revealed that health-related behaviors are influenced by partner behaviors (43). Sleep durations were more favorable among older adults with spouses or partners than among single older adults. In married individuals, most of whom sleep in the same bed as their spouse, sleep is influenced by their spouse’s breathing, snoring, and waking (44). Thus, sensory impairment may be influenced by marital status.

We found that persistently short or long sleep duration and changes from normal to short or long sleep duration markedly affected the risk of sensory impairment in older people aged 65–84 years but not in those aged ≥85 years. This difference may be explained by several physiological and psychological factors. Younger older adults are typically in the early stages of retirement; they tend to have insomnia, anxiety, or depression if they fail to adapt to their reduced work capacity and altered lifestyle and health status. Both anxiety and depression serve as risk factors for sensory impairment (30).

## 5 Strengths and limitations

To the best of our knowledge, this study is the first to investigate the effects of altered sleep duration on sensory impairment in Chinese older adults by using nationally representative cohort data. Our study has some limitations. Firstly, this study used the 2008–2014 data on sleep duration. Although sleep duration and altered sleep duration in this study is similar with other recent studies (45, 46), older adults might have a downward trend in sleep duration over the past 13 years (47), and an increased prevalence of short sleep duration (48). The change in sleep duration indicates that more attention should be paid to the impact of abnormal sleep duration on health of older adults. Secondly, we used self-reported sleep data; thus, the possibility of a recall bias cannot be ignored. Nonetheless, a good agreement has been demonstrated between self-reported sleep data and objectively measured sleep data (49, 50). Furthermore, several items on the sensory impairment questionnaire that participants completed lacked objective parameters; therefore, the questionnaire responses might have been biased. Objective data on sleep and sensory impairment are needed to further clarify the effects of altered sleep duration on sensory impairment. Thus, in the future, researchers should obtain sleep data through wrist actigraphy and polysomnography and confirm VI and HI diagnoses by using relevant data obtained under the guidance of a medical professional.

## 6 Conclusion

A sleep duration of ≥9 h is associated with an increased risk of sensory impairment. Compared with persistently normal sleep duration, persistently short or long sleep duration and changes from normal to short or long sleep duration are associated with an elevated risk of sensory impairment. Irregular sleep patterns and sensory disorders are becoming increasingly common in older adults. Our findings highlight the need for improving sleep duration and establishing healthy sleep habits to reduce the risk of sensory disorders in older adults, particularly educated and younger older adults. Thus, community health-care providers should offer sleep health education to older adults, thereby increasing their awareness regarding healthy sleep. Targeted interventions are needed to address persistently short or long sleep duration in this population.

## Data availability statement

The original contributions presented in the study are included in the article/supplementary material, further inquiries can be directed to the corresponding author.

## Author contributions

LY: Data curation, Formal analysis, Writing – original draft. JD: Software, Validation, Visualization, Writing – original draft. YD: Conceptualization, Methodology, Project administration, Writing – review & editing. YC: Data curation, Validation, Writing – original draft. QQ: Data curation, Investigation, Writing – original draft. ZL: Methodology, Validation, Writing – review & editing. HL: Conceptualization, Funding acquisition, Resources, Supervision, Writing – review & editing.
